# The other side of cardiac Ca^2+^ signaling: transcriptional control

**DOI:** 10.3389/fphys.2012.00452

**Published:** 2012-11-28

**Authors:** Alejandro Domínguez-Rodríguez, Gema Ruiz-Hurtado, Jean-Pierre Benitah, Ana M. Gómez

**Affiliations:** ^1^Inserm U769, IFR141, Labex Lermit, University of Paris-Sud 11Châtenay-Malabry, France; ^2^Unidad de Hipertensión, Hospital Universitario 12 de Octubre, Instituto de Investigación Sanitaria i+12, and Instituto Pluridisciplinar, Universidad ComplutenseMadrid, Spain

**Keywords:** heart, calcium, excitation-transcription coupling, TRPC, nuclear calcium

## Abstract

Ca^2+^ is probably the most versatile signal transduction element used by all cell types. In the heart, it is essential to activate cellular contraction in each heartbeat. Nevertheless Ca^2+^ is not only a key element in excitation-contraction coupling (EC coupling), but it is also a pivotal second messenger in cardiac signal transduction, being able to control processes such as excitability, metabolism, and transcriptional regulation. Regarding the latter, Ca^2+^ activates Ca^2+^-dependent transcription factors by a process called excitation-transcription coupling (ET coupling). ET coupling is an integrated process by which the common signaling pathways that regulate EC coupling activate transcription factors. Although ET coupling has been extensively studied in neurons and other cell types, less is known in cardiac muscle. Some hints have been found in studies on the development of cardiac hypertrophy, where two Ca^2+^-dependent enzymes are key actors: Ca^2+^/Calmodulin kinase II (CaMKII) and phosphatase calcineurin, both of which are activated by the complex Ca^2+^/Calmodulin. The question now is how ET coupling occurs in cardiomyocytes, where intracellular Ca^2+^ is continuously oscillating. In this focused review, we will draw attention to location of Ca^2+^ signaling: intranuclear ([Ca^2+^]_n_) or cytoplasmic ([Ca^2+^]_c_), and the specific ionic channels involved in the activation of cardiac ET coupling. Specifically, we will highlight the role of the 1,4,5 inositol triphosphate receptors (IP_3_Rs) in the elevation of [Ca^2+^]_n_ levels, which are important to locally activate CaMKII, and the role of transient receptor potential channels canonical (TRPCs) in [Ca^2+^]_c_, needed to activate calcineurin (Cn).

Ca^2+^ has evolved as the most versatile signal transduction pathway used by all cells (Berridge et al., 2000), but perhaps no other cell type uses Ca^2+^ in such different ways as cardiac myocytes do, in normal physiology and as a major contributor to heart disease. First evidenced by Ringer as the signal carrier initiating contraction (Ringer, 1883), Ca^2+^ is known to control other key cardiac cell processes (Berridge et al., 1998) including initiation of pacemaker activity, action potential (AP) shape, regulation of cell–cell communication, arrhythmogenesis, metabolism, and transcriptional regulation. All these processes use Ca^2+^ as a nexus, which auto controls its own cellular fluxes, as illustrated by the Ca^2+^-induced Ca^2+^ release mechanism (Fabiato, 1983) underlying excitation-contraction (EC) coupling, as well as the Ca^2+^-induced Ca^2+^-entry (Richard et al., 2006) participating in excitation-transcription (ET) coupling. ET coupling is the process by which signaling molecules that regulate EC-coupling activate Ca^2+^-dependent transcription factors (Anderson, 2000). In the adult heart, neurohormonal/mechanical stress enhances ET coupling, resulting in cell growth (hypertrophy), reexpression of the fetal gene program, and alteration of ionic channels and transporter expression (Chevalier et al., 1989; Marbán and Koretsune, 1990; Chien et al., 1991; Moalic et al., 1993; Gidh-Jain et al., 1995; Nass et al., 2008). The transcription factors involved in cardiac hypertrophy have been reviewed by Heineke and Molkentin (Heineke and Molkentin, 2006). Among them, myocyte enhancer factor 2 (MEF2) and GATA4 are initiated by a cascade activated by Ca^2+^/Calmodulin (CaM): CaM Kinase II (CaMKII) for MEF2 (Passier et al., 2000) and calcineurin (Cn) for GATA4 (Molkentin et al., 1998; Houser and Molkentin, 2008).

Thus, Ca^2+^ activates contraction in the heart in a beat-to-beat fashion, while it is also able to activate hypertrophy by ET coupling at a longer time scale (Maier and Bers, 2002). The mechanisms by which the heart differentiates between Ca^2+^ signals are only beginning to be elucidated. In this review, we will focus on the implication of local pools of Ca^2+^ in activating gene transcription in adult ventricular cardiomyocytes, as during hypertrophy development.

## Ca^2+^ signaling in ventricular myocytes

Ca^2+^ is a key element in cardiac EC coupling. In each heartbeat, membrane depolarization during an AP activates L-type Ca^2+^ channels (LTCCs) located in the sarcolemma. Ca^2+^ entry activates intracellular Ca^2+^ release channels, named ryanodine receptors (RyRs), located in the membrane of the sarcoplasmic reticulum (SR). RyRs amplify the initial Ca^2+^ signal, providing enough Ca^2+^ to activate contractile myofibrils. Relaxation occurs when cytosolic Ca^2+^ concentration ([Ca^2+^]_c_) returns to diastolic values, due mainly to Ca^2+^ pumped back into the SR by the Ca^2+^-ATPase (SERCA) and extrusion from the cell via the Na^+^/Ca^2+^ exchanger (Bers, 2002). New roles for intracellular Ca^2+^ ([Ca^2+^]_i_) are being elucidated (Bers, 2008). For instance, prohypertrophic transcription factors are activated by nuclear/perinuclear activation of CaMKII promoted by local elevation of nuclear [Ca^2+^] ([Ca^2+^]_n_) (Wu et al., 2006): CaMKII phosphorylates histone deacetylases (HDAC) 4 and 5, resulting in their translocation out of the nucleus, derepriming the transcription factor MEF2. Cytoplasmic Ca^2+^ elevations are also involved in ET coupling by activating Cn, which dephosphorylates the nuclear factor of activated T cells (NFAT), which is imported into the nucleus where it activates the transcription factor GATA (Molkentin et al., 1998).

However, it is still not fully understood whether [Ca^2+^]_n_ variations can be dissociated from bulk [Ca^2+^]_i_ oscillations during contraction-relaxation cycles. The proposed mechanisms are the location and the specificity of the channels. Thus, rapid elevations in cytoplasmic Ca^2+^ activate contraction, while [Ca^2+^]_n_ activates Ca^2+^-dependent transcription factors. Regarding the channels and oversimplifying the situation: if Ca^2+^ comes from SR, the channel involved in contractile activity is the RyR, while the one involved in transcription is the inositol 1,4,5 triphosphate receptor (IP_3_R). The location, RyR in the SR and IP_3_R in the nuclear envelope (NE) and perinuclear area, preferentially affects cytosolic and [Ca^2+^]_n_, respectively. When Ca^2+^ enters through the sarcolemma, the specific channel involved may also help to differentiate contractile *vs*. transcriptional Ca^2+^. LTCCs are mainly involved in contraction, while other less known Ca^2+^ permeating channels in the cardiomyocyte, such as TRPCs, play an important role in hypertrophy development (Wu et al., 2010). However, LTCCs may also be involved in transcription activation. It has been shown that the C-terminal part of LTCCs may travel from the membrane to the nucleus, activating transcription. The T-type Ca^2+^ channels have been shown to be involved in cell growth. However, in the adult myocyte this channel is not or is only very weakly expressed. At late stages of Ca^2+^ hypertrophy, the T-type Ca^2+^ channels are reexpressed (Nuss and Houser, 1993; Martinez et al., 1999), but their implication in the initiation of hypertrophy has not been demonstrated.

Below we summarize some of the known aspects of transcription induction by [Ca^2+^]_n_, focusing on the role of IP_3_R, and by [Ca^2+^]_c_, focusing on the role of TRPCs. The involvement of two Ca^2+^-dependent enzymes, Cn and CaMKII, has been established. Their involvement in cardiac hypertrophy-ET coupling is reviewed in Bers (2008) and Molkentin (2000), among others.

## Nuclear Ca^2+^ in ET coupling

The question of how ET coupling can co-exist in cardiac myocytes in which [Ca^2+^]_c_ continuously oscillates within each heartbeat remains a matter of debate. Localization of the Ca^2+^ signal restricted to microdomains may be the answer. It has thus been postulated that intranuclear/perinuclear Ca^2+^ is involved in ET coupling, whereas [Ca^2+^]_c_ is responsible for EC coupling. While there is no doubt on the second, whether or not [Ca^2+^]_n_ signaling is independently regulated from cytosolic Ca^2+^ is not that clear. In fact, the NE [which also acts as a Ca^2+^ reservoir, continuously to the SR (Wu and Bers, 2006)] has pores permeable to Ca^2+^ (Bootman et al., 2009). Thus, [Ca^2+^]_c_ can passively diffuse into the nucleus, challenging the possibility of an independence of [Ca^2+^]_n_ from cytosolic [Ca^2+^]_c_. This important question is still not answered. However, the hypothesis of separately controlled domains is supported by the following: **(1)** the location of Ca^2+^ release channels is different in SR and NE; **(2)** some molecules preferentially affect [Ca^2+^]_n_; and **(3)** [Ca^2+^]_n_ signal decay is slower, due mainly to the lack of SERCA in the inner membrane of the NE (Bootman et al., 2009), and thus under conditions of fast pacing Ca^2+^ can be accumulated in the nucleoplasm initiating the hypertrophic signaling.

1. The location of RyRs on the junctional SR, facing LTCCs (located on the T-tubules), is crucial for EC coupling in ventricular myocytes. Other Ca^2+^ release channels expressed in cardiac myocytes are the IP_3_Rs, which are concentrated on the NE/perinuclear area (Escobar et al., 2011). After activation of Gq-coupled protein receptors, phospholipase C (PLC) is activated, producing IP_3_. Activation of IP_3_Rs provide Ca^2+^ to the intranuclear or perinuclear region where activate local CaMKII, which phosphorylates class II HDAC, prompting their translocation out of the nucleus and derepressing the prohypertrophic transcription factor MEF2 (McKinsey et al., 2000; Zhang et al., 2002). IP_3_Rs are also expressed at the junctional SR of hypertrophied hearts, where they may play a role in EC coupling (Harzheim et al., 2009) under this pathological condition. Furthermore, RyRs may also be expressed in the NE (Bootman et al., 2009), although its role there is not known.
2. Some prohypertrophic molecules have shown an action elevating [Ca^2+^]_n_ more than [Ca^2+^]_c_. For instance, endothelin, which activates Gq and PLC producing IP_3_, increases [Ca^2+^]_n_ in both atrial (Kockskamper et al., 2008a,b) and ventricular myocytes (Wu et al., 2006) independently of [Ca^2+^]_c_. Recently, we analyzed the effects on [Ca^2+^]_n_ of Epac (De Rooij et al., 1998), a protein with prohypertrophic actions in cardiac myocytes (Morel et al., 2005; Metrich et al., 2008). This protein is directly activated by cAMP and contributes to β-adrenergic-induced cardiac hypertrophy (Metrich et al., 2008). Epac induces IP_3_ production (Metrich et al., 2010; Pereira et al., 2012) and a significant increase in [Ca^2+^]_n_, correlating with the perinuclear expression pattern of Epac (Pereira et al., 2012). Moreover, sustained Epac activation (from 30 min) drives the HDAC5 nuclear export in a manner that is CaMKII- and IP_3_Rs-dependent, with the consequent activation of MEF2 (Metrich et al., 2010; Pereira et al., 2012).
3. Oscillating Ca^2+^ may also be an important contributor to the activation of gene transcription. Increasing the frequency of [Ca^2+^]_i_ transients (as in tachycardia) induces cardiac hypertrophy and heart failure (HF). It is not known whether the cell is stimulated by an increase in the time-average [Ca^2+^]_i_ or if, because [Ca^2+^]_n_ dynamics are slower than cytoplasmic ones, there is an accumulation of Ca^2+^ in the nucleoplasm at higher frequencies.

## Cytoplasmic Ca^2+^ in ET coupling

Although nuclear localization is involved in ET coupling, mathematical models have predicted that separate compartments may not be necessary *in vitro* (Cooling et al., 2009). Without disregarding the relevance of [Ca^2+^]_n_ in ET coupling, [Ca^2+^]_c_ may also play a role. In fact, Ca^2+^/CaM activates Cn, found in the cytosol, which is involved in hypertrophy (Molkentin et al., 1998). When activated, Cn dephosphorylates NFAT in the cytoplasm, permitting its translocation to the nucleus where it participates in the hypertrophic gene expression (Heineke and Molkentin, 2006). Moreover, the plasma membrane Ca^2+^ ATPase antagonizes Ca^2+^ hypertrophy, suggesting that extruding Ca^2+^ from the cytosol, probably close to Cn, prevents its activation (Wu et al., 2009).

The Ca^2+^ entry pathways which may activate Cn are being elucidated. LTCCs located in lipid rafts could form a Ca^2+^ signaling microdomain (Houser and Molkentin, 2008). But other Ca^2+^-permeable channels may be located on these microdomains to activate Cn. Ca^2+^ entry through TRPC channels is necessary to induce hypertrophy (Wu et al., 2010). Most of the TRPC studies have been conducted in non-excitable cells, and thus their role in ventricular myocytes is not yet completely clear, although the proof that they are needed for cardiac hypertrophy has highlighted an important role in the heart (Wu et al., 2010). Ca^2+^ influxes through LTCCs and TRPCs are thus the proximal sources of Ca^2+^ influx that regulate cardiac gene expression in adult ventricular cells. These Ca^2+^ influxes might influence gene expression by several mechanisms. Ca^2+^ can diffuse to the nucleus and activate nuclear calcium-dependent transcription factors and coregulators (Hardingham et al., 2001) or Ca^2+^ can activate calcium-dependent signaling proteins around the mouth of the channel, which propagate the signal to the nucleus (Deisseroth et al., 1998; Dolmetsch et al., 2001). Another mechanism was recently observed in neurons (Gomez-Ospina et al., 2006) and cardiac myocytes (Schroder et al., 2009). The C-terminal domain of the LTCC pore-forming subunit, Cav1.2, might be truncated as a result of post-translational processing. The cleaved fragment, in a Ca^2+^-dependent manner, translocates to the nucleus and acts as a transcription factor to control the transcription of a variety of genes, including Cav1.2.

### L-type Ca^2+^ channels (LTCCs)

Treating myocardial cultures with high potassium to inhibit spontaneous contractions (and LTCCs) results in decreased myosin and ribosomal RNA expression (McDermott et al., 1985, 1991; Samarel and Engelmann, 1991). In neonatal rat ventricular cell cultures, LTCC activators stimulate atrial natriuretic factor (ANF) expression (Sei et al., 1991), and ANF expression induced by electrical stimulation of contractions was inhibited by nifedipine, an LTCC blocker (McDonough and Glembotski, 1992). Moreover, Zn^2+^ influx via voltage-dependent Ca^2+^ channels can turn on gene expression (Atar et al., 1995). Similarly to what was previously described in skeletal muscle cells (Taouis et al., 1991; Duff et al., 1992), treatment with verapamil, a Ca^2+^ channel blocker, increases the Na^+^ channel α-subunit mRNA levels in neonatal rat cardiac myocytes, while treatment with A23187, a Ca^2+^ ionophore, leads to a decrease in the mRNA levels (Chiamvimonvat et al., 1995). In adult ventricular myocytes, transient changes in [Ca^2+^]_i_ can modulate Cav1.2 mRNA and protein abundance, producing a corresponding change in functional Ca^2+^ channels (Davidoff et al., 1997). Surprisingly, whereas early studies in mammalian heart muscle were unable to detect an increased number of channels (Nishiyama et al., 1986; Gengo et al., 1988), an LTCC block by *in vivo* pharmacological treatment might result in up-regulation of L-type Ca^2+^ current (I_Ca,L_), Ca_V_1.2 protein, and mRNA (Chapados et al., 1992; Chiappe De Cingolani et al., 1994; De Cingolani et al., 1996; Morgan et al., 1999; Schroder et al., 2007). We found some lines of evidence supporting this hypothesis. We saw that aldosterone, a neurohormone involved in HF, (1) activates LTCC expression (Bénitah and Vassort, 1999), (2) increases diastolic Ca^2+^ release by decreasing the expression of the RyR accessory proteins FKBP12 and 12.6 (Gomez et al., 2009), and (3) decreases the expression of the channel responsible for the transient outward potassium current (*I*_to_) secondarily to an increase in [Ca^2+^]_i_ and activation of Cn (Bénitah et al., 2003; Perrier et al., 2004), thereby recapitulating some of the outcomes of HF (Bénitah et al., 1993, 2002; Gómez et al., 1997; Marx et al., 2000). Interestingly, the increase in LTCC expression precedes cell hypertrophy (Perrier et al., 2003).

There is evidence that physiopathological perturbations in Ca_V_1.2 Ca^2+^ influx regulate K^+^ channel expression. We have seen that aldosterone increases LTCC expression (Bénitah and Vassort, 1999), which secondarily decreases the expression of the channel responsible for *I*_to_ (Bénitah et al., 2003). Consistently, we have reported that increased Ca^2+^ influx results in decreased *I*_to_ density, as a result of down-regulation of Kv4.2 transcript expression mediated by Cn (Perrier et al., 2004). Although it has been reported that expression of a constitutively active form of Cn increases *I*_to_ densities through the up-regulation of Kv4.2 transcript expression in neonatal rat ventricular myocytes (Gong et al., 2006), the transcriptional down-regulation of Kv4.2 across the ventricular wall (Rossow et al., 2006), as well as following myocardial infarction (Rossow et al., 2004), results from differences in [Ca^2+^]_i_ that appear to underlie a differential activation of Cn and NFAT. In addition, it has been reported that increased CaMKII activity down-regulates Kv4.3 transcript expression, resulting in decreased *I*_to_ densities in isolated canine ventricular myocytes (Xiao et al., 2008).

Thus in cardiac myocytes, although not as broadly illustrated in other cell types (Barbado et al., 2009), it clearly appears that Ca^2+^ itself, or even other divalent cations like Zn^2+^ influx through LTCCs, is involved in transcriptional regulation and/or post-transcriptional events in response to membrane depolarization. This is of particular importance but it is not always taken into account in acquired or inherited cardiac diseases, during which AP duration is altered.

Although LTCCs have been the focus of the majority of the studies with regard to non-cardiac and cardiac gene regulation, some studies also suggest the implication of Ca^2+^ entry through non-L-type channels in ET coupling, notably TRPC channels.

### TRPC channels

TRPC channels provide Ca^2+^ entry pathways, modulate the driving force for Ca^2+^ entry, and also likely provide intracellular pathways for Ca^2+^ release from cellular organelles. Preferentially localized to the peripheral plasma membrane in cardiomyocytes (Kuwahara et al., 2006; Seth et al., 2009; Wu et al., 2010), they are cation-selective channels that initiate cardiac hypertrophy by Ca^2+^ influx and subsequent Cn activation (Bush et al., 2006; Kuwahara et al., 2006; Nakayama et al., 2006; Onohara et al., 2006).

The TRPC family includes 7 isoforms (TRPC1–7) divided into 2 general subfamilies based on structural and functional similarities: TRPC1/4/5 and TRPC3/6/7. TRPC2 is not expressed in humans (Lof et al., 2011). TRPC channels can be homomeric or heteromeric assemblies between 4 TRPC subunits. Each TRPC subunit has a transmembrane region flanked by functionally important intracellular N and C termini (Clapham, 2003). TRPC3/6/7 are activated by diacylglycerol (DAG) generated by G-protein coupled receptors Gαq/PLC signaling. TRPC1/4/5 can be activated by depletion of intracellular Ca^2+^ stores or by stretch (Nilius et al., 2007; Abramowitz and Birnbaumer, 2009). Once activated, these channels induce signal transduction through elevations in [Ca^2+^]_i_ and Na^+^ or through refilling of ER Ca^2+^ stores to ensure prolonged signaling events (Nilius et al., 2007; Abramowitz and Birnbaumer, 2009).

**Figure 1:**
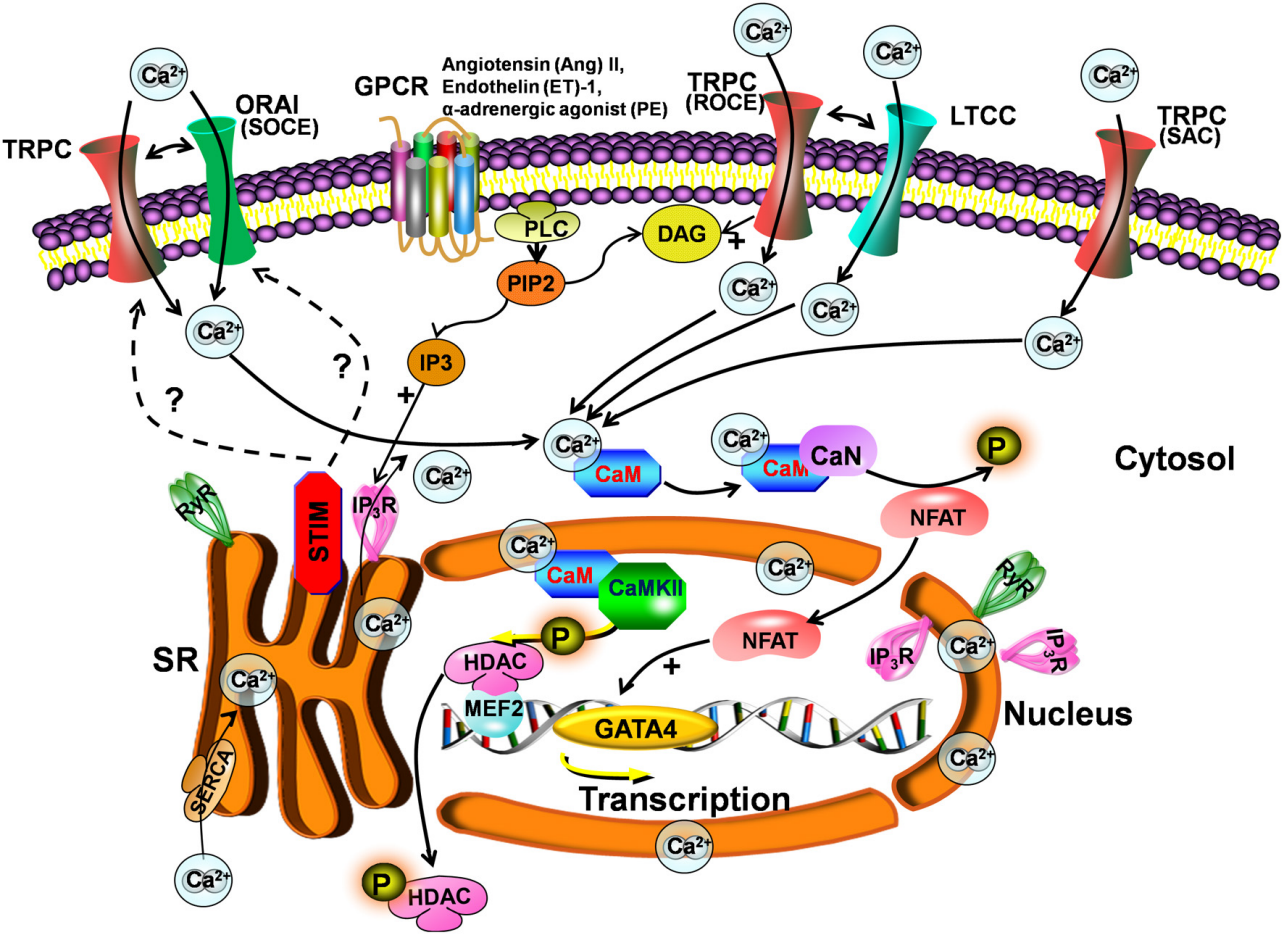
**Scheme for the TRPC signaling pathway in hypertrophy.** Stimulation of Gq-protein coupled receptors (GPCR) and subsequent activation of PLC leads to IP_3_ and DAG generation. DAG directly activates TRPCs and induces receptor-operated Ca^2+^ entry (ROCE), causing membrane depolarization and secondarily activating LTCCs. IP_3_ activates IP_3_Rs, which induce depletion of stores and activation of STIM. STIM1 provokes store-operated Ca^2+^ entry (SOCE) through Orai and/or TRPCs. TRPC channels can also be activated by stretch (SAC). The increase in intracellular Ca^2+^ following TRPC activation is involved in hypertrophy development via activation of the calcineurin-NFAT pathway.

One controversy surrounding TRPC channels concerns their participation in store-operated Ca^2+^ entry (SOCE) versus receptor-operated Ca^2+^ entry (ROCE) (Figure 1). TRPC1/4/5 channels are proposed candidate subunits of store-operated channels (SOCs). These types of channels are activated by IP_3_-dependent mechanisms (Nishida et al., 2006). TRPC3/6/7 are directly activated by DAG, independently of the stores (Hofmann et al., 1999) linked to PLC activation. TRPC channels might also sense and transduce mechanical stress (stretch-activated Ca^2+^ channels, Figure 1). Another study suggested that TRPC3/6 are activated by DAG causing membrane depolarization with effects on LTCCs and Ca^2+^ oscillations (Onohara et al., 2006) (Figure 1).

The role of TRPC channels in SOCE is less clear since the discovery of stromal interaction molecule 1 (STIM1) and Orai1 as mediators of SOCE. STIM1 serves as a Ca^2+^ sensor in the endoplasmic reticulum/SR, which, when is Ca^2+^ depleted, clusters proximal to the plasma membrane to activate Orai1, the pore-forming subunit of the Ca^2+^ release-activated channel (Frischauf et al., 2008) but possibly also to activate TRPC channels (Figure 1). Indeed, it has been shown that TRPC1/4/5 can directly bind STIM1, activating SOCE (Yuan et al., 2007). STIM1 can also indirectly activate TRPC3/6, but not TRPC7 (Liao et al., 2009). Interestingly, TRPC channels can also colocalize with STIM1 and Orai in lipid raft domains (Pani et al., 2008). One study even suggests that Orai and TRPC form complexes that participate in SOCE and ROCE (Liao et al., 2009). However, other investigators have not observed a role for TRPC channels in the Orai/STIM1 complex, suggesting a model whereby these 2 mechanisms of Ca^2+^ entry are distinct and not coregulated (Dehaven et al., 2009). Interestingly, STIM1 amplifies agonist-induced hypertrophy via activation of the Cn-NFAT pathway (Luo et al., 2012). Figure 1 summarizes some of the TRPC pathways involved in ET coupling.

In conclusion, [Ca^2+^]_i_, besides its major role in EC coupling, is an important messenger in signal transduction regulating cardiac hypertrophy by activation of Ca^2+^-dependent transcription factors. Here we have attempted to present some of the pathways by which cardiac Ca^2+^ signaling is involved in ET coupling, notably during cardiac hypertrophy development. Although the profound influence of Ca^2+^ signaling on gene expression has been recognized mainly in neurons (Dolmetsch, 2003), the notion of cardiac ET coupling has recently emerged (Atar et al., 1995; Anderson, 2000; Richard et al., 2006). Evidence is growing that intracellular signaling pathways are laid down in a very sophisticated manner to enable cardiac cells to distinguish between Ca^2+^ signals. This is particularly important during cardiac hypertrophy, which occurs in response to a variety of stimuli (neurohumoral stimulation, stretch, and pacing) but is initiated in many cases by an elevation in [Ca^2+^]_i_. New discoveries are expected in the near future on cardiac Ca^2+^ regulation to further enrich our understanding in this fascinating research field.

### Conflict of interest statement

The authors declare that the research was conducted in the absence of any commercial or financial relationships that could be construed as a potential conflict of interest.
